# Impact of Outdated Clinical and Laboratory Standards Institute (CLSI) Breakpoint Implementation on Antimicrobial Susceptibility Interpretation: A Retrospective Analytical Study

**DOI:** 10.7759/cureus.104338

**Published:** 2026-02-26

**Authors:** Nisha Goyal, Seema Gangar, Bhavya Ramakrishnan, Monika Goma, Gayathri SJ, Shukla Das

**Affiliations:** 1 Microbiology, University College of Medical Sciences, Delhi, IND; 2 Microbiology, Maharishi Markandeshwar Institute of Medical Sciences & Research, Mullana, IND

**Keywords:** amr, breakpoints, clsi, resistance underreporting, revised ast cutoff, update guidelines

## Abstract

Introduction

Using outdated breakpoints can lead to a considerable overestimation of susceptibility, which can result in ineffective treatments; therefore, it's crucial to adopt current standards to improve patient outcomes.

Methods

This retrospective analytical study analyzed 9,279 bacterial isolates. Reporting errors in antimicrobial susceptibility testing (AST) by the Kirby-Bauer disk diffusion method due to the use of outdated breakpoints was assessed by comparing the revised cutoff breakpoints of specific antibiotics with previous Clinical and Laboratory Standards Institute (CLSI) guidelines. Chi-square testing and McNemar’s test were used to evaluate paired interpretive shifts between the older and updated CLSI breakpoints.

Results

Among *Enterobacterales*, analysis of 2,262 aminoglycoside zone diameters revealed significant misclassification when outdated CLSI breakpoints were applied. Gentamicin also showed a significant shift in susceptibility distribution (χ² = 95.27, p < 0.0001), with paired analysis confirming correction of susceptible isolates to the intermediate category using updated breakpoints (McNemar χ² = 36.0, p < 0.0001). Amikacin demonstrated a borderline overall shift (χ² = 6.00, p = 0.0499) but substantial paired misclassification (McNemar χ² = 36.0, p < 0.0001). In *Pseudomonas aeruginosa*, piperacillin-tazobactam (χ² = 6.62, p = 0.0366) and tobramycin (χ² = 77.94, p < 0.0001) demonstrated a significant redistribution of susceptibility categories, with older criteria overestimating susceptibility. Among *Staphylococcus aureus*, implementation of revised 34th-edition CLSI linezolid breakpoints introduced an intermediate category and resulted in significant reclassification from susceptible to intermediate (χ² = 17.45, p = 0.00016; McNemar χ² = 17.0, p < 0.0001).

Conclusion

Unregulated and delayed implementation of updated CLSI breakpoints results in significant misclassification of antimicrobial susceptibility, leading to overestimation of susceptibility and masking emerging resistance. Standardized and timely adoption of revised breakpoints, along with supportive laboratory systems, is essential to ensure accurate susceptibility interpretation, effective antimicrobial stewardship, and reliable antimicrobial resistance surveillance.

## Introduction

Antimicrobial susceptibility testing (AST) of clinical isolates is one of the most important functions of a clinical microbiology laboratory. AST of a pathogen plays a crucial role in advising clinicians on therapy choices and monitoring the prevalence of antimicrobial resistance (AMR) [1]. The Clinical and Laboratory Standards Institute (CLSI) M100 performance standards are widely used in many countries including India for the interpretation of AST results, which are performed by disk diffusion (i.e., Kirby-Bauer method) or minimum inhibitory concentration (MIC) based methods. Many clinical laboratories still rely on disk diffusion as the primary AST method, and these guidelines enable the microbiologists to interpret the results as susceptible, intermediate, or resistant for a given antibiotic by comparing it with these standard guidelines [2].

The CLSI interpretive cutoffs are established on the basis of pharmacokinetic-pharmacodynamic (PK-PD) properties, MIC distributions, and the mechanisms of resistance in antibiotics [1]. The World Health Organization (WHO) recommends the methods described by CLSI and EUCAST (European Committee on Antimicrobial Susceptibility Testing) for surveillance [3]. Indian Council of Medical Research (ICMR) recommends the use of CLSI guidelines for the surveillance of the antimicrobial susceptibility pattern [4].

The CLSI annually updates the M100 document. The breakpoint revision is a complicated and dynamic process, which is needed from time to time; therefore, there is a need for integrating the most recent microbiological resistance pattern, PK-PD, and clinical outcome, as the previous breakpoints might not be able to accurately predict the treatment efficacy of the tested antimicrobials. It is necessary to understand that the use of an obsolete breakpoint is more likely to result in poor patient outcomes. To impact therapy choices at an institutional level, implementation of current breakpoints should be a priority [5,6]. In CLSI (M100-Edition 33), the major changes focus on the selective & cascade reporting of antibiotics and comments related to therapeutics. The CLSI has evolved from its classical approach of grouping the antibiotics (A, B, U, O) to the current tier-based approach (Tier 1, 2, 3, 4) that has implications in suggesting cascade reporting of the antibiotics [2,7].

The breakpoints of aminoglycosides (gentamicin, amikacin, tobramycin) for *Enterobacterales *and *Pseudomonas aeruginosa* (*P. aeruginosa*) and linezolid for Staphylococcal species have been updated. Aminoglycosides are potent, broad-spectrum antibiotics and remain one of the most prescribed antimicrobial agents in almost all bacterial infections. These antimicrobials are active against various gram-positive and gram-negative bacteria. They act via inhibition of protein synthesis and can synergize with other antibacterial classes, thus considered for combination therapy [8]. In case of *P. aeruginosa*, gentamicin has been removed, and the testing of amikacin has been restricted for urinary isolates only [7]. The breakpoints of piperacillin-tazobactam are also updated for *P. aeruginosa*. Piperacillin-tazobactam is a potent β-lactam/β-lactamase inhibitor (BL/BLIs), commonly administered in critically ill patients for the management of serious infections caused by *P. aeruginosa *[9,10].

Linezolid is the first marketed representative of the oxazolidinone class effective against drug-resistant gram-positive bacterial infections, such as methicillin-resistant *Staphylococcus aureus* (MRSA), penicillin-resistant *Streptococcus*, and vancomycin-resistant* Enterococcus* (VRE) as well as for drug-resistant tuberculosis as the bedaquiline-pretomanid-linezolid regimen. Therefore, linezolid, being an effective choice for treating multidrug and extensive drug-resistant tuberculosis (MDR-TB/XDR-TB), should be used even more cautiously [11-13].

In terms of absolute volume, India tops the global consumption of antibiotics [14]. Self-medication, over-the-counter availability of antibiotics in the absence of prescriptions and evidence of bacterial infection, further contributes to AMR [14,15]. At the microbiologist’s end, under-reporting of resistance due to unregulated implementation of revised AST breakpoints can lead to inadvertent use of inappropriate antibiotics. Failure to update CLSI breakpoints in many clinical laboratories can be attributed to multiple operational and technical barriers. These include outdated automated system software, reliance on vendor-supplied manuals, and dependence on instrument platforms whose built-in breakpoints cannot be modified manually. Many laboratories continue to use older interpretive criteria because vendor software updates are delayed or unavailable. The high annual cost of updated CLSI documents also presents a significant barrier, particularly for laboratories in low- and middle-income countries (LMICs) [16]. Limited awareness and underutilization of WHONET (World Health Organization Network) software may further contribute to the problem; although WHONET is free, its installation and effective use require training and basic computer infrastructure. This tool is especially valuable for AMR surveillance and preparing local antibiograms in peripheral or rural laboratories with or without automated systems, yet many laboratories remain unaware that WHONET can automatically update CLSI breakpoints [16,17]. Additional challenges include limited staff training, administrative constraints, Laboratory Information Systems (LISs) that are incompatible with new categories such as SDD (susceptible dose-dependent) or NS (non-susceptible), revised zone diameter cutoffs, and the absence of strong antimicrobial stewardship oversight [16]. All these factors contribute to a distorted understanding of the actual antibiotic resistance patterns [18,19]. This potentially delays appropriate interventions, impacts treatment outcomes, and contributes to the spread of resistant strains of bacteria. Furthermore, inaccurate data can hinder effective public health measures and the development of targeted strategies to combat antibiotic resistance. This retrospective analytical study assessed the impact of using outdated CLSI breakpoints on routine AST, highlighting how delayed adoption of updated standards leads to significant overestimation of susceptibility. 

## Materials and methods

This retrospective analytical study was conducted over a duration of one and a half years, from January 2023 to June 2024, at the bacteriology laboratory of a tertiary care hospital in Delhi, India. Ethical approval was waived by the institutional ethical committee due to the retrospective nature of the study, with no patient involvement or identifiable data. The samples of pus aspirate, lower respiratory tract, genital specimens and sterile body fluids received in our laboratory for routine culture and AST were included in this study. After removing duplicate isolates and contaminants, a total of 9,279 bacterial isolates were included in the analysis. Clinical specimens were inoculated onto Blood agar and MacConkey agar (HiMedia Laboratories) for primary isolation of pathogens. Following culture growth, isolates were identified using standard laboratory procedures based on conventional biochemical tests. AST was subsequently carried out using the Kirby-Bauer disc diffusion method on Mueller-Hinton agar with antibiotic discs, both procured from HiMedia Laboratories. The zones of inhibition were interpreted as per the latest CLSI guidelines [2,20,21]. For the interpretation of errors in the reporting of AST resulting from the use of outdated CLSI, the zones of inhibition of gentamicin (10 µg), amikacin (30µg), tobramycin (10µg) for *Enterobacterales*; tobramycin (10µg) and piperacillin-tazobactam (100/10µg) for *P. aeruginosa*; and linezolid (30µg) for Staphylococcal species were analyzed using the latest CLSI guidelines, and the raw zone diameters were reanalysed and were compared against the previous year’s CLSI guidelines (Table 1) [2,20,21]. Antibiotic testing was guided by cascade reporting, organism and sample relevance, and availability; therefore, not all agents were tested for every isolate. Quality control testing was carried out daily using the reference strains *Escherichia coli* ATCC 25922, Pseudomonas aeruginosa ATCC 27853, and *Staphylococcus aureus* ATCC 25921 as a part of routine laboratory procedures [2,20,21]. Statistical analysis was done by using IBM SPSS Statistics for Windows, Version 31 (Released 2025; IBM Corp., Armonk, New York, United States). Chi-square analysis was used to describe overall differences in the distribution of susceptibility categories (susceptible, intermediate/SDD, resistant) between older and updated CLSI breakpoint versions. McNemar’s test was used as the primary analysis to evaluate paired categorical changes, as the same isolates were reassessed under both CLSI criteria. A p-value < 0.05 was considered statistically significant. 

**Table 1:** Revised antimicrobial breakpoints for the disk diffusion method according to CLSI M100 guidelines*. Zone diameter breakpoints are expressed in millimeters (mm); S: Susceptible; I: Intermediate; R: Resistant; SDD: Susceptible, dose-dependent. Breakpoints are based on Clinical and Laboratory Standards Institute (CLSI M100) editions corresponding to the respective years. “No change in breakpoints” indicates no revision in CLSI interpretive criteria during the specified year. Disk content is shown in parentheses. *[2,20,21]

Antimicrobial disk (disk content)	2022 (CLSI M100-Ed32)				2023 (CLSI M100-Ed33)				2024 (CLSI M100-Ed34)			
Enterobacterales	S	SDD	I	R	S	SDD	I	R	S	SDD	I	R
Gentamicin (10 µg)	≥15	-	13-14	≤12	≥18	-	15-17	≤12	No change in breakpoints			
Amikacin (30 µg)	≥17	-	15-16	≤14	≥20	-	17-19	≤16				
Tobramycin (10 µg)	≥15	-	13-14	≤12	≥17	-	13-16	≤12				
Pseudomonas aeruginosa												
Piperacillin- Tazobactam (100/10 µg)	≥21	15-20	-	≤14	≥22	18-21	-	≤17	No change in breakpoints			
Tobramycin (10 µg)	≥15	-	13-14	≤12	≥19	-	13-18	≤12				
Staphylococcal species												
Linezolid (30 µg)	No change in breakpoints				≥21	-	-	≤20	≥26	-	23-25	≤22

## Results

**Figure 1:**
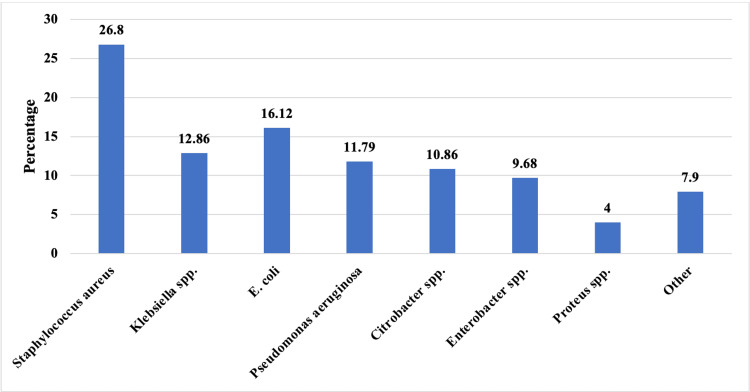
Distribution of bacterial isolates over the study period (N= 9279).

A total of 9,279 clinical isolates were recovered during the study period, comprising 3,219 gram-positive cocci (GPC) (34.7%) and 6,060 gram-negative bacilli (GNB) (65.3%). Among the GNB, *Enterobacterales* accounted for 53.5% (4966) of all pathogens. *S. aureus* was the most prevalent organism (n = 2489; 26.8%), followed by *E. coli *(n = 1,496; 16.1%), Klebsiella spp. (n = 1,193; 12.9%), *Pseudomonas aeruginosa *(n = 1,094; 11.8%), Citrobacter spp. (n = 1,008; 10.9%), Enterobacter spp. (n = 898; 9.7%), and Proteus spp. (n = 371; 4.0%). The remaining isolates (n=730; 7.9%) comprised other GPC (e.g., Enterococcus spp., Streptococcal spp., coagulase-negative staphylococci) and other non-fermenters like Acinetobacter spp. The distribution of common bacteria isolated over the study period is depicted in Figure 1. 

Following the application of updated CLSI breakpoints, notable shifts in antimicrobial susceptibility interpretations were observed. The breakpoints of gentamicin (10µg), amikacin (30µg), and tobramycin (10µg) for *Enterobacterales* and tobramycin (10µg) and piperacillin-tazobactam (100/10µg) for *P. aeruginosa* were revised in 2023 (CLSI M100 33rd edition) [2]. In 2024 (CLSI M100 34th edition), breakpoints of linezolid (30µg) for Staphylococcal species were revised (Table 1) [19,21,22].

Among Enterobacterales, 2262 zone diameters were assessed for aminoglycosides. A total of 1433 isolates were tested for susceptibility to gentamicin, 584 for amikacin, and 245 for tobramycin in the year 2023. The interpretation of aminoglycoside zone diameters categorized as susceptible, intermediate, and resistant according to the older (2022) and updated (2023) CLSI guidelines is presented in Table 2 [2,21].

**Table 2:** Comparison of antimicrobial susceptibility interpretations using older and updated CLSI breakpoints with Chi-square analysis. N: total number of isolates tested for respective antimicrobial, S: Susceptible; I: Intermediate; R: Resistant; * SDD: Susceptible, dose-dependent instead if intermediate; CLSI: Clinical and Laboratory Standards Institute χ²: Chi-square test; p-value < 0.05 considered statistically significant

Isolates	Antibiotic	N	Older CLSI			Updated CLSI			χ²	p-value
			S	I	R	S	I	R		
Enterobacterales	Gentamicin	1433	686	14	733	567	120	746	95.27	<0.0001
	Amikacin	584	178	30	376	142	38	404	6.00	0.0499
	Tobramycin	245	70	13	162	66	17	162	0.65	0.722
Pseudomonas aeruginosa	Piperacillin–Tazobactam*	450	326	47*	77	318	31*	101	6.62	0.0366
	Tobramycin	529	326	14	189	240	100	189	77.94	<0.0001
Staphylococcus aureus	Linezolid	333	331	0	2	314	17	2	17.45	0.00016

Table 2 compares the AST interpretations using older and updated CLSI breakpoints with Chi-square analysis. For *Enterobacterales*, both Chi-square and McNemar analyses demonstrated noticeable misclassification of aminoglycoside susceptibility when outdated CLSI breakpoints were used. Gentamicin showed the most prominent change, with a highly significant shift in overall susceptibility distribution (χ² = 95.27, p < 0.0001). Paired analysis confirmed that using the updated CLSI breakpoints significantly improved accuracy, correctly shifting isolates to the intermediate category that older criteria had misclassified as susceptible (McNemar χ² = 36.0, p < 0.0001). 

Amikacin showed a borderline significant shift in susceptibility categories (χ² = 6.00, p = 0.0499). However, on paired analysis, amikacin showed substantial misclassification with 36 isolates that were intermediate under the updated breakpoints being incorrectly reported as susceptible when older CLSI criteria were used (McNemar χ² = 36.0, p < 0.0001). 

For tobramycin, overall susceptibility proportions did not differ significantly between the two breakpoint versions (χ² = 0.65, p = 0.722). However, paired analysis still revealed misclassification, with a small number of isolates falsely categorized as susceptible by older breakpoints but correctly interpreted as intermediate under the updated CLSI criteria (McNemar χ² = 4.0, p = 0.0455).

Among non-fermenters, 1094 (11.8%) were *P. aeruginosa*. The susceptibility patterns of piperacillin-tazobactam (100/10mg) and tobramycin (10mg) were interpreted as per CLSI M100- E32nd & Ed 33rd edition and are given in Table 2 [2,21]. Notably, the CLSI guidelines has SDD category for piperacillin-tazobactam instead of the intermediate category. Piperacillin-tazobactam showed a statistically significant change in susceptibility pattern distribution (χ² = 6.62, p = 0.0366). Paired comparison further highlighted interpretive errors associated with the older criteria, as eight isolates originally categorized as susceptible were reassigned to the SDD using the revised breakpoints (McNemar χ² = 8.0, p = 0.0046). Tobramycin exhibited an even more substantial shift, with a marked redistribution across susceptibility categories (χ² = 77.94, p < 0.0001). Paired analysis identified major misclassification of 86 isolates, which were classified as susceptible by the older criteria, were correctly placed in the intermediate category under the updated guidelines (McNemar χ² = 86.0, p < 0.0001).

Among the S. aureus isolates (26.8%), linezolid susceptibility testing was performed for 333 isolates in 2024. The 2024 CLSI M100, 34th edition introduced revised breakpoints and added an intermediate category for linezolid for the first time [19]. The updated breakpoints resulted in a significant redistribution of susceptibility categories (χ² = 17.45, p = 0.00016), primarily driven by the introduction of a newly defined intermediate category, which accounted for 17 isolates previously classified as susceptible. The paired McNemar’s test also demonstrated a significant reclassification from susceptible to intermediate (χ² = 17.0, p < 0.0001), indicating that the updated criteria substantially increase the proportion of isolates categorized as intermediate [19].

## Discussion

AST breakpoints are critical for accurate result interpretation and are regularly revised by CLSI to incorporate emerging resistance patterns, clinical outcomes, and PK/PD data [4]. Although CLSI guidelines remain widely used, many laboratories continue to rely on outdated CLSI breakpoints for several reasons. Annual CLSI updates require paid access which can pose a financial burden, particularly for laboratories in LMICs [1]. In contrast, EUCAST guidelines are freely available, making them an attractive alternative from a cost perspective. However, implementation of EUCAST is not straightforward, as substantial practical and methodological differences exist between CLSI and EUCAST, such as variations in breakpoints, testing conditions, and interpretive criteria. As highlighted by previous studies, although EUCAST has been advocated as a preferred AST system in LMICs, the challenges associated with transitioning from CLSI should not be underestimated. Such transitions may result in marked changes in institutional antibiograms due to breakpoint discrepancies, potentially affecting clinical decision-making and complicating AMR surveillance. These challenges ultimately underscore the importance of implementing updated CLSI guidelines as early as possible to prevent overlooked resistance and inaccurate susceptibility reporting [1,5,6].

Our findings clearly demonstrate that non-implementation of updated CLSI breakpoints leads to significant misclassification of bacterial susceptibility, thereby masking true resistance levels. These discrepancies can undermine AMR control efforts by distorting the actual resistance landscape. Together, these observations highlight the critical need for laboratories to regularly adopt updated laboratory standards to ensure accurate and reliable AST reporting.

Several older CLSI guideline editions used higher susceptibility breakpoints, resulting in a greater proportion of isolates being reclassified as susceptible compared with revised criteria. As per the 2019 revision of CLSI, the ciprofloxacin and levofloxacin breakpoints had a notable impact on routine AST performance, particularly for isolates with MICs near the revised cutoffs. Disk diffusion demonstrated reduced categorical agreement, most prominently for ciprofloxacin, with an increased rate of minor errors largely attributable to isolates being classified as more resistant than by broth microdilution. Levofloxacin disk diffusion performed comparatively better and met CLSI acceptability criteria following error rate bound analysis. While the E-test showed excellent essential agreement with the reference method for both agents, categorical agreement was lower, especially for levofloxacin, with frequent minor errors arising from single-dilution MIC differences around the breakpoint. These findings highlight the inherent variability of susceptibility testing near interpretive thresholds and emphasize the need for cautious interpretation of results close to breakpoints in clinical laboratories [16,22,23].

In regions with high and rising antibiotic consumption, particularly LMICs, accurate breakpoint implementation is of even greater importance. In our study, application of updated CLSI breakpoints for *Enterobacterales* revealed significant misclassification of aminoglycoside susceptibility, particularly for gentamicin, which showed frequent false susceptibility under older criteria. Amikacin also demonstrated significant misclassification on paired analysis, despite only borderline changes in overall proportions, while tobramycin showed minimal overall variation, although a few isolates were still incorrectly classified as susceptible. These discrepancies are clinically relevant, regardless of sample size, and illustrate how reliance on outdated guidelines can misrepresent true susceptibility patterns. Our observations are consistent with previous studies on MDR and carbapenem-resistant *Enterobacterales* (CRE), which reported reductions in susceptibility to amikacin, gentamicin, and tobramycin following application of revised CLSI breakpoints. One such study documented a decline in amikacin susceptibility from 75% to 59% in CRE, from 96% to 79% in ESBL producers, and from 94% to 68% in MDR isolates [24]. However, other studies have reported higher amikacin susceptibility rates, highlighting variability across settings and reinforcing the need for standardized, up-to-date interpretive criteria [25].

Combination therapy with BL/BLIs, such as piperacillin-tazobactam, and aminoglycosides remains central to the management of serious infections caused by *P. aeruginosa* [25]. The inclusion of the SDD category enhances AST reporting by providing a more nuanced assessment of antimicrobial activity. Clinically, SDD interpretation guides optimized dosing strategies and prevents unnecessary exclusion of potentially effective agents. Misclassification of SDD isolates as susceptible under older CLSI breakpoints may lead to suboptimal dosing and treatment failure, whereas resistant isolates misidentified as SDD may result in unwarranted dose escalation and toxicity [16]. In this study, significant reclassification was observed for piperacillin-tazobactam and tobramycin highlights how outdated breakpoints can distort susceptibility interpretation and adversely affect clinical decision-making in P. aeruginosa infections.

Similar effects of breakpoint revisions have been reported previously. Kuo et al. demonstrated a marked reduction in piperacillin-tazobactam susceptibility among *Enterobacterales *following adoption of CLSI 2022 breakpoints, with susceptibility decreasing from 80.9% to 50%. Other studies have shown that failure to update CLSI breakpoints can result in substantial misclassification of susceptibility, while revised breakpoints for tobramycin in P. aeruginosa have similarly led to reduced susceptibility rates, particularly among isolates previously categorized as susceptible [26,27].

Another example that emphasizes the limitations of unregulated breakpoints is the concept of nonsusceptibility (NS). An NS result does not necessarily indicate a defined resistance mechanism but reflects an MIC exceeding the susceptible range, often due to limited clinical outcome data. This approach improves clinical decision-making and helps reduce treatment failures, as observed by increasing reports of daptomycin NS and associated therapeutic failures in *Enterococcus* species [16,17].

Linezolid is widely used for severe infections caused by *S. aureus* and *Enterococcus* spp., including pneumonia, skin and soft tissue infections, and sepsis. The excellent oral bioavailability and intravenous availability of linezolid have contributed to its widespread use and potential overprescription [11,12]. Accurate interpretation of linezolid susceptibility is therefore essential to prevent inappropriate utilization. Despite the small number of linezolid-resistant isolates and overall low resistance rates in our study, application of updated CLSI breakpoints resulted in a statistically significant redistribution of susceptibility categories, primarily due to the introduction of a new intermediate category. This reclassification underscores the clinical relevance of adopting revised interpretive criteria, even when resistance prevalence is low.

The limitations of this study include the single-center design, use of disk diffusion only, lack of clinical outcome correlation and absence of economic impact analysis. Nevertheless, our findings clearly demonstrate significant discrepancies in the interpretation of AST. The updated breakpoints can be more readily implemented using disk diffusion and gradient diffusion methods than automated MIC systems. Although automated AST platforms are widely used, their fixed antimicrobial and MIC dilution ranges per AST panels necessitate reliance on commercial vendors for panel and software updates, thereby delaying adoption of revised guidelines. In such settings, until automated AST systems, including testing cards and associated software, are updated, disk diffusion or gradient diffusion methods should be promptly employed for antimicrobials with revised breakpoints. Additionally, the use of WHONET software can facilitate accurate AMR surveillance and antibiogram generation by applying updated interpretive criteria, thereby supporting timely and reliable susceptibility reporting [16,17,27].

## Conclusions

Outdated CLSI breakpoints result in clinically significant misclassification of antimicrobial susceptibility, masking true resistance and thus compromising antimicrobial stewardship and AMR surveillance. This study showed significant discrepancies in AST interpretation of aminoglycosides among *Enterobacterales*, with gentamicin demonstrating the most pronounced shift in susceptibility distribution and amikacin exhibiting borderline change. In *P. aeruginosa*, piperacillin-tazobactam and tobramycin showed significant redistribution of susceptibility categories, while implementation of the revised 34th-edition CLSI breakpoints led to significant reclassification of linezolid from susceptible to intermediate among *Staphylococcus* species. Timely implementation of revised breakpoints is therefore essential. Increased awareness, improved software support, and systematic guideline compliance are necessary to strengthen laboratory practice and resistance monitoring. Until automated AST systems are updated, disk diffusion or gradient diffusion methods, supported by WHONET-based surveillance, provide a practical and reliable interim approach to ensure accurate susceptibility reporting.

## References

[1] Cusack TP, Ashley EA, Ling CL, Roberts T, Turner P, Wangrangsimakul T, Dance DA (2019). Time to switch from CLSI to EUCAST? A Southeast Asian perspective. Clin Microbiol Infect.

[2] Wayne PA (2023). Clinical and Laboratory Standards Institute (CLSI). Performance Standards for Antimicrobial Susceptibility Testing.

[3] (2024). The European Committee on Antimicrobial Susceptibility Testing. Breakpoint tables for interpretation of MICs and zone diameters. Version 13.0,2023. http://www.eucast.org.

[4] Nabadda S, Kakooza F, Kiggundu R (2021). Implementation of the World Health Organization global antimicrobial resistance surveillance system in Uganda, 2015-2020: mixed-methods study using national surveillance data. JMIR Public Health Surveill.

[5] Kronvall G, Giske CG, Kahlmeter G (2011). Setting interpretive breakpoints for antimicrobial susceptibility testing using disk diffusion. Int J Antimicrob Agents.

[6] Walia K, Madhumathi J, Veeraraghavan B (2019). Establishing antimicrobial resistance surveillance & research network in India: journey so far. Indian J Med Res.

[7] Humphries RM, Abbott AN, Hindler JA (2019). Understanding and addressing CLSI breakpoint revisions: a primer for clinical laboratories. J Clin Microbiol.

[8] Krause KM, Serio AW, Kane TR, Connolly LE (2016). Aminoglycosides: an overview. Cold Spring Harb Perspect Med.

[9] Fawaz S, Barton S, Nabhani-Gebara S (2020). Comparing clinical outcomes of piperacillin-tazobactam administration and dosage strategies in critically ill adult patients: a systematic review and meta-analysis. BMC Infect Dis.

[10] Tannous E, Lipman S, Tonna A, Hector E, Hussein Z, Stein M, Reisfeld S (2020). Time above the MIC of piperacillin-tazobactam as a predictor of outcome in Pseudomonas aeruginosa bacteremia. Antimicrob Agents Chemother.

[11] Swaney SM, Aoki H, Ganoza MC, Shinabarger DL (1998). The oxazolidinone linezolid inhibits initiation of protein synthesis in bacteria. Antimicrob Agents Chemother.

[12] Dresser LD, Rybak MJ (1998). The pharmacologic and bacteriologic properties of oxazolidinones, a new class of synthetic antimicrobials. Pharmacotherapy.

[13] Conradie F, Bagdasaryan TR, Borisov S (2022). Bedaquiline-pretomanid-linezolid regimens for drug-resistant tuberculosis. N Engl J Med.

[14] Koya SF, Ganesh S, Selvaraj S, Wirtz VJ, Galea S, Rockers PC (2022). Antibiotic consumption in India: geographical variations and temporal changes between 2011 and 2019. JAC Antimicrob Resist.

[15] Bimba HV, Roy V, Batta A, Daga MK (2020). Drug utilization, rationality, and cost analysis of antimicrobial medicines in a tertiary care teaching hospital of Northern India: a prospective, observational study. Indian J Pharmacol.

[16] Abbey TC, Deak E (2019). What's new from the CLSI subcommittee on antimicrobial susceptibility testing M100. Clinical Microbiol Newslett.

[17] Stelling J, O'Brien TF (2016). WHONET: software for surveillance of infecting microbes and their resistance to antimicrobial agents. Molecular Microbiology: Diagnostic Principles and Practice.

[18] Humphries R, Bobenchik AM, Hindler JA, Schuetz AN (2021). Overview of changes to the clinical and laboratory standards institute performance standards for antimicrobial susceptibility testing, M100. J Clin Microbiol.

[19] Nassar MS, Hazzah WA, Bakr WM (2019). Evaluation of antibiotic susceptibility test results: how guilty a laboratory could be?. J Egypt Public Health Assoc.

[20] Wayne PA (2024). Clinical and Laboratory Standards Institute. Performance Standards for Antimicrobial Susceptibility Testing. https://drive.google.com/file/d/1oHs-hMxvCQw_Bg_0IE9n87UoIVkdQYAV/view.

[21] Wayne PA (2022). Clinical and Laboratory Standards Institute. Performance Standards for Antimicrobial Susceptibility Testing. 2022CLSI M100-S32. https://clsi.org/about/news/clsi-publishes-m100-performance-standards-for-antimicrobial-susceptibility-testing-32nd-edition/.

[22] Humphries RM, Hindler JA, Shaffer K, Campeau SA (2019). Evaluation of ciprofloxacin and levofloxacin disk diffusion and Etest using the 2019 Enterobacteriaceae CLSI breakpoints. J Clin Microbiol.

[23] Yarbrough ML, Wallace MA, Potter RF, D'Souza AW, Dantas G, Burnham CD (2020). Breakpoint beware: reliance on historical breakpoints for Enterobacteriaceae leads to discrepancies in interpretation of susceptibility testing for carbapenems and cephalosporins and gaps in detection of carbapenem-resistant organisms. Eur J Clin Microbiol Infect Dis.

[24] Sader HS, Mendes RE, Kimbrough JH, Kantro V, Castanheira M (2023). Impact of the recent clinical and laboratory standards institute breakpoint changes on the antimicrobial spectrum of aminoglycosides and the activity of plazomicin against multidrug-resistant and carbapenem-resistant Enterobacterales from United States Medical Centers. Open Forum Infect Dis.

[25] Walkty A, Adam H, Baxter M, Lagace-Wiens P, Karlowsky J, Zhanel G (2023). 2813. Impact of a recent change in tobramycin breakpoints (CLSI) on the proportion of Pseudomonas aeruginosa clinical isolates that are multidrug-resistant: CANWARD, 2016 to 2021. Open Forum Infect Dis.

[26] Kuo S (2023). A-280 Impact of 2022 CLSI revised piperacillin/tazobactam clinical breakpoints on Enterobacterales isolates identified at a tertiary medical center in southern Taiwan. Clin Chem.

[27] Humphries RM, Ambler J, Mitchell SL (2018). CLSI methods development and standardization working group best practices for evaluation of antimicrobial susceptibility tests. J Clin Microbiol.

